# Left ventricular hypertrophy in experimental chronic kidney disease is associated with reduced expression of cardiac Kruppel-like factor 15

**DOI:** 10.1186/s12882-018-0955-9

**Published:** 2018-07-03

**Authors:** Sheila K. Patel, Elena Velkoska, Daniel Gayed, Jay Ramchand, Jessica Lesmana, Louise M. Burrell

**Affiliations:** Department of Medicine, Austin Health, The University of Melbourne, Level 7 Lance Townsend Building, Austin Hospital, 145 Studley Road, Heidelberg, VIC 3084 Australia

**Keywords:** KLF15, Kruppel-like factor 15, Renin angiotensin system, Left ventricular hypertrophy, ACE inhibition, Subtotal nephrectomy

## Abstract

**Background:**

Left ventricular hypertrophy (LVH) increases the risk of death in chronic kidney disease (CKD). The transcription factor Kruppel-like factor 15 (KLF15) is expressed in the heart and regulates cardiac remodelling through inhibition of hypertrophy and fibrosis. It is unknown if KLF15 expression is changed in CKD induced LVH, or whether expression is modulated by blood pressure reduction using angiotensin converting enzyme (ACE) inhibition.

**Methods:**

CKD was induced in Sprague–Dawley rats by subtotal nephrectomy (STNx), and rats received vehicle (*n* = 10) or ACE inhibition (ramipril, 1 mg/kg/day, *n* = 10) for 4 weeks. Control, sham-operated rats (*n* = 9) received vehicle. Cardiac structure and function and expression of KLF15 were assessed.

**Results:**

STNx caused impaired kidney function (*P* < 0.001), hypertension (*P* < 0.01), LVH (*P* < 0.001) and fibrosis (*P* < 0.05). LVH was associated with increased gene expression of hypertrophic markers, atrial natriuretic peptide (ANP), brain natriuretic peptide (BNP, *P* < 0.01) and connective tissue growth factor (CTGF) (*P* < 0.05). Cardiac KLF15 mRNA and protein expression were reduced (*P* < 0.05) in STNx and levels of the transcription regulator, GATA binding protein 4 were increased (*P* < 0.05). Ramipril reduced blood pressure (*P* < 0.001), LVH (*P* < 0.001) and fibrosis (*P* < 0.05), and increased cardiac KLF15 gene (*P* < 0.05) and protein levels (*P* < 0.01). This was associated with reduced ANP, BNP and CTGF mRNA (all *P* < 0.05).

**Conclusion:**

This is the first evidence that loss of cardiac KLF15 in CKD induced LVH is associated with unchecked trophic and fibrotic signalling, and that ACE inhibition ameliorates loss of cardiac KLF15.

## Background

Cardiovascular (CV) disease is the leading cause of death in patients with chronic kidney disease (CKD) [1]. Left ventricular hypertrophy (LVH) which is characterised by myocyte hypertrophy and interstitial fibrosis [2] is highly prevalent in CKD [3] and independently predicts sudden cardiac death [4, 5]. Although blood pressure reduction [6] can regress LVH [7], LVH can also progress despite treatment [8] and thus remains a major cause of adverse CV outcomes [3, 9].

An improved understanding of the molecular mechanisms of CKD induced LVH may lead to new therapeutic targets. Our group is interested in the potential role of the transcription factor Kruppel-like factor 15 (KLF15), a member of a large family of zinc finger transcription factors [10] which is highly expressed in adult cardiac myocytes and fibroblasts [11–13]. The significance of KLF15 in human disease was illustrated by our recent paper which identified a variant in the *KLF15* gene relevant to the development of LVH in patients with type 2 diabetes [14].

Experimentally, both in vitro and in vivo studies support a role for KLF15 as a repressor of pathological cardiac hypertrophy and fibrosis [11–13] which occurs through inhibition of the activity of two pivotal pro-hypertrophic transcriptional regulators, GATA binding protein 4 (GATA4) and myocyte enhancer factor 2 (MEF2) and their effects on the atrial natriuretic peptide (ANP) and brain natriuretic peptide (BNP) promoters [11].

To date the role of KLF15 in CKD induced LVH is unknown. In this paper, we investigate the expression of cardiac KLF15 in an experimental model of kidney disease induced by subtotal nephrectomy (STNx) [15]. We previously reported that STNx leads to LVH, interstitial fibrosis, an activated cardiac renin angiotensin system (RAS) and impaired cardiac function; angiotensin converting enzyme (ACE) inhibition partially ameliorated these effects [16, 17]. We hypothesised that LVH in CKD would be associated with: (1) deficiency of cardiac KLF15, increased expression of its transcriptional regulators (GATA4, MEF2), and increased expression of hypertrophic and pro-fibrotic markers (ANP, BNP and connective tissue growth factor (CTGF)); and (2) that ACE inhibition would reverse LVH and fibrosis in CKD and restore cardiac KLF15 levels and corresponding changes in transcriptional regulators and hypertrophic and pro-fibrotic markers.

## Methods

### Experimental protocol

Female Sprague–Dawley rats (body weight 200–225 g) were purchased from Monash University (Melbourne, Australia) and were housed at 23–25 °C in a 12 h:12 h light–dark cycle, with ad libitum food containing 0.4–0.6% NaCl (Norco, NSW, Australia) and water. Rats were weighed and allocated to one of three groups (Control, STNx vehicle or ramipril) according to body weight in order to ensure the mean body weight per group was similar pre-operatively. There were no significant differences in pre-operative body weight between the 3 groups (mean ± SEM, Control (201 ± 3.7 g); STNx-vehicle (207 ± 1.3 g); STNx- ramipril (211 ± 2.3 g), *P* = 0.163). One animal in the Control group died unexplained prior to sham surgery. There were no further adverse events. In brief, rats were anesthetized by an intraperitoneal injection of pentobarbitone sodium (60 mg/kg/body weight, Boehringer Ingelheim, NSW, Australia), and STNx (*n* = 20) or sham surgery (Control, *n* = 9) was performed as described previously [15–17] by removal of the right kidney, and infarction of two-thirds of the left kidney. Following surgery, STNx rats received daily vehicle (*n* = 10) or the ACE inhibitor, ramipril (1 mg/kg/day, *n* = 10) by gavage for 28 days. Sham-operated Control rats received vehicle (*n* = 9). Investigators were not blinded to the disease groups or treatment. However, the cardiac assessment was performed by an investigator who was blinded to both model and treatment, and all biochemical, gene expression and histological analysis were performed in a blinded manner.

On day 27, rats were housed in metabolic cages and a urine sample collected. On day 28, rats were anaesthetised with intraperitoneal sodium pentobarbitone (60 mg/kg/body weight), and cardiac haemodynamics were assessed as previously described [15]. Heart rate, systolic and diastolic blood pressure, mean arterial pressure (MAP), LV end-diastolic pressure (LVEDP) and the maximal and minimal rate of ventricular contraction (d*P*/d*t*_max,_ d*P*/d*t*_min_) and the time constant of isovolumic relaxation (Tau) were measured. Rats were killed by a lethal dose of sodium pentobarbitone, decapitated, trunk blood collected, centrifuged, and plasma snap-frozen and stored at -80 °C. The heart and left kidney was removed and weighed, and the left ventricle (LV) was transversely dissected with one piece fixed in 4% paraformaldehyde and embedded in paraffin for histopathology, and the remainder snap frozen in isopentane and stored at -80 °C for mRNA extraction.

### Biochemical analysis

Plasma and urine creatinine and plasma urea were measured using an autoanalyser (Beckman Instruments, Palo Alto, CA, USA). Urinary protein was determined using the bicinchoninic acid (BCA) method with a commercially available Pierce™ BCA protein assay kit (ThermoFisher Scientific, Victoria, Australia). Plasma ACE activity was measured as previously described [18].

### Cardiac (LV) collagen and KLF15 immunohistochemistry

Collagen was assessed as previously described [15–17] using LV (4 μm) sections which were deparaffinized, rehydrated, and stained with 0.1% Sirius Red (Polysciences Inc) in saturated picric acid (picrosirius red) for 1 h, differentiated in 0.01% HCl for 30 s, and rapidly dehydrated. Interstitial collagen volume fraction was determined by measuring the area of stained tissue within a given field, excluding vessels, artefacts, minor scars or incomplete tissue fields; 15–20 fields were analysed per animal (*n* = 6/gp). The area stained was calculated as a percentage of the total area within a given field.

Immunohistochemical staining for cardiac KLF15 (polyclonal antibody, Aviva, Sapphire Bioscience, Australia, diluted 1:100) was performed in LV (4 μm) sections. Sections were deparaffinized, rehydrated, and then endogenous peroxidase activity quenched for 30 min by incubation in 3% H_2_O_2_ in methanol. The primary antibody was applied and left to incubate overnight at 4 °C. The Elite Vectastain ABC kit (Vector Laboratories, CA, USA) followed by 3,3-Diaminobenzidine (Sigma, St Louis, MO, USA) were used to visualize antibody binding. Slides were counterstained with haematoxylin and cover-slipped. Negative control slides were incubated with normal goat serum in the absence of the primary antibody. Staining was quantified using computerized image analysis (MCID, Imaging Research, Ontario, Canada). Images were imported into the AIS Imaging program (AIS Imaging, Ontario, Canada) using a colour video camera and a standard light microscope (magnification × 20). The detection level threshold for positively stained areas (brown for 3,3-Diaminobenzidine staining) was set so that the processed image accurately reflected the positively stained areas as visualized by light microscopy on the unprocessed digital image. KLF15 staining was quantitated by measuring the area of stained tissue within a given field, excluding vessels, artifacts, minor scars or incomplete tissue fields. Fifteen fields were analysed per animal (*n* = 6/gp). The density x area of staining was determined by calculating the number of selected pixels (positively stained areas) in the total area within a given field.

### Cardiac (LV) gene expression

Total RNA was isolated from LV using the RNeasy kit method (Qiagen). cDNA was synthesized with a reverse transcriptase reaction using standard techniques (Superscript III kit; Life Technologies) as described previously [15]. Rat cardiac KLF15, ANP, BNP, CTGF, GATA4 and MEF2c gene expression was determined using TaqMan assays (ThermoFischer Scientific, Victoria, Melbourne). TaqMan gene expression assay identifiers used were as follows: KLF15 - Rn00585508_m1; BNP - Rn04219558_g1; ANP - Rn00664637_g1; CTGF - Rn01537279_g1; GATA4 - Rn01530459_m1 and MEF2A - Rn01478096_m1). Cardiac ACE gene expression was determined using primers and probes as previously described [19]. Quantitative RT-PCR was performed using the TaqMan system based on real-time detection of accumulated fluorescence (7500 Real Time PCR System, Applied Biosystems, CA, USA). Gene expression was normalized to 18S VIC and reported as the fold change compared with expression in vehicle treated Control rats which were given an arbitrary value of one.

### Statistics

Data are shown as mean ± standard error of mean (SEM) and were analysed using SPSS (version 22, IBM). Independent samples T-test was used to compare the difference between Control and STNx rats to determine the effect of CKD on KLF15. Vehicle-treated STNx rats were compared to STNx rats treated with ramipril to determine the effect of ACE inhibition on cardiac KLF15 in rats with CKD. The Spearmans rho was determined for the relationship between KLF15 gene and protein expression and LV mass using the data from Control and STNx-vehicle rats. Two-tailed *P* values < 0.05 were considered significant.

## Results

### Physiological and biochemical effects of STNx

At 4 weeks after STNx, rats had reduced body weight (*P* < 0.05) and kidney impairment with elevated plasma creatinine (*P* < 0.001) and plasma urea, proteinuria (*P* < 0.01) and reduced creatinine clearance (*P* < 0.001) compared to Control rats (Table 1). In STNx rats, treatment with ramipril reduced plasma ACE activity (< 0.0001) and proteinuria (*P* < 0.05) but did not change plasma creatinine and urea or creatinine clearance.

**Table 1:** Body weight, plasma markers and kidney function

	Control	Subtotal nephrectomy (STNx)	
	Vehicle	Vehicle	Ramipril
n	9	10	10
Body weight (g)	232 ± 3.6	223 ± 1.6*	241 ± 4.2^††^
Left kidney weight (g)	0.75 ± 0.09	1.15 ± 0.07***	1.01 ± 0.51^†††^
Left kidney /body weight (g/100 g)	0.24 ± 0.01	0.35 ± 0.01***	0.24 ± 0.003^†††^
Plasma ACE activity (nmol/ml/hr)	1474 ± 156	1624 ± 101	13 ± 4^†††^
Plasma urea (mmol/l)	5.3 ± 0.2	9.5 ± 0.6***	10.8 ± 0.7
Urine protein (mg/100 g/24 h)	57.2 ± 2.5	91.7 ± 10.7**	66.6 ± 3.2^†^
Plasma creatinine (μmol/l)	22 ± 1	45 ± 4***	46 ± 2
Creatinine clearance (ml/min)	2.84 ± 0.20	1.39 ± 0.17***	1.49 ± 0.10

Data are presented as mean ± SEM. Control vs. STNx-vehicle (disease effect) *P < 0.05, **P < 0.01, ***P < 0.001; STNx-vehicle vs. ramipril (treatment effect) ^†^*P* < 0.05, ^††^*P* < 0.01, ^†††^*P* < 0.001

*Abbreviations:* *ACE* Angiotensin converting enzyme

### Blood pressure and cardiac function

STNx rats had significant and marked increases in systolic and diastolic blood pressure (Table 2, *P* < 0.01) and MAP (*P* < 0.001). CKD led to adverse effects on cardiac function with hypercontractility (*P* < 0.05), and diastolic dysfunction with impaired active relaxation (Tau, *P* < 0.05), and increased LVEDP (*P* < 0.01). Ramipril had significant cardiovascular benefits to decrease blood pressure, Tau and LVEDP (all *P* < 0.01) (Table 2).

**Table 2:** Blood pressure and cardiac function and fibrosis

	Control	Subtotal nephrectomy (STNx)	
	Vehicle	Vehicle	Ramipril
n	9	10	10
Blood pressure and cardiac function			
Heart rate (beats/min)	354 ± 10	377 ± 17	380 ± 12
Systolic blood pressure (mmHg)	139 ± 4	205 ± 18**	119 ± 6^†††^
Diastolic blood pressure (mmHg)	108 ± 3	138 ± 11**	90 ± 6^††^
Mean arterial pressure (mmHg)	125 ± 4	189 ± 6***	106 ± 6^†††^
d*P*/d*t*_max_ (mmHg/s)	10,870 ± 776	14,739 ± 1468*	11,342 ± 924
d*P*/d*t*_min_ (mmHg/s)	−10,593 ± 710	−10,570 ± 1509	−10,556 ± 836
Tau (ms)	14 ± 1	18 ± 1*	13 ± 1^††^
LVEDP (mmHg)	10.8 ± 0.9	17.1 ± 1.8**	11.4 ± 0.7^†^
Cardiac fibrosis			
Interstitial (%)	2.5 ± 0.33	8.9 ± 1.8*	3.6 ± 0.2^†^
Perivascular (%)	23 ± 2	27 ± 2	28 ± 2

Data are presented as mean ± SEM. Control vs. STNx-vehicle (disease effect) *P < 0.05, **P < 0.01, ***P <0.001; STNx-vehicle vs. ramipril (treatment effect) ^†^*P* < 0.05, ^††^*P* < 0.01, ^†††^*P* < 0.001

*Abbreviations:* *LVDEP* left ventricular end diastolic pressure

### Cardiac hypertrophy and fibrosis

**Fig. 1:**
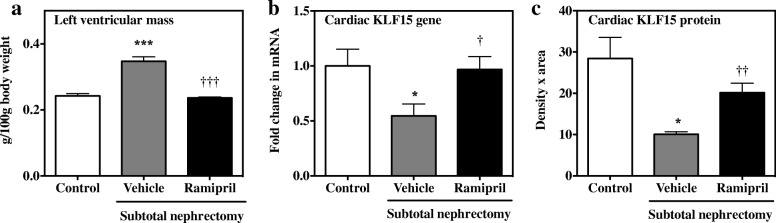
Left ventricular mass and KLF15 gene and protein expression. **a** Change in left ventricular mass (g/100 g body weight) **b** Fold change in KLF15 mRNA relative to Control group; **c** KLF15 protein quantitated by immunohistochemistry. Data are presented as mean ± SEM. Control vs. subtotal nephrectomy -Vehicle (disease effect) *P < 0.05, ***P < 0.001; subtotal nephrectomy -Vehicle vs. ramipril (treatment effect) ^†^*P* < 0.05, ^††^*P* < 0.01, ^†††^*P* < 0.001

STNx rats had LV hypertrophy (*P* < 0.001, Fig. 1a) and interstitial fibrosis (*P* < 0.05, Table 2) compared to Control rats. In STNx rats, ACE inhibition reduced LV mass (*P* < 0.001, Fig. 1a) and interstitial fibrosis (*P* < 0.05, Table 2) compared to vehicle treatment.

### Cardiac (LV) gene expression

Changes in LV gene expression with STNx and the effect of ramipril are shown in Table 3. Cardiac ACE gene expression was unchanged in STNx rats compared to Control, and reduced by ramipril (*P* < 0.01). STNx rats had a significant increase in gene expression of markers of cardiac hypertrophy (ANP, BNP, both *P* < 0.01). Expression of ANP and BNP is regulated by pro-hypertrophic transcriptional factors including MEF2A and GATA4, and STNx rats had increased expression of GATA4 mRNA (*P* < 0.05), but no change in MEF2A. STNx rats also had increased cardiac expression of the pro-fibrotic marker, CTGF (*P* < 0.05). Ramipril reduced ACE gene expression, ANP (*P* < 0.01), BNP (*P* < 0.05) and CTGF (*P* < 0.01) but had no effect on GATA4 or MEF2A mRNA expression. Ramipril had no effect on either GATA4 or MEF2A mRNA expression in STNx rats.

**Table 3:** Left ventricular gene expression

Fold change in mRNA (arbitrary units)	Control	Subtotal nephrectomy (STNx)	
	Vehicle	Vehicle	Ramipril
n	9	10	10
Angiotensin converting enzyme	1.00 ± 0.09	0.81 ± 0.08	0.38 ± 0.06^††^
Atrial natriuretic peptide	1.00 ± 0.26	7.98 ± 2.09**	0.92 ± 0.25^††^
Brain natriuretic peptide	1.00 ± 0.17	2.97 ± 0.55**	1.16 ± 1.22^†^
Connective tissue growth factor	1.00 ± 0.17	7.76 ± 0.29*	0.48 ± 0.17^††^
GATA binding protein 4	1.00 ± 0.09	1.83 ± 0.3*	1.17 ± 0.18
Myocyte enhancer factor 2A	1.00 ± 0.11	1.64 ± 0.29	1.14 ± 0.18

Data are presented as mean ± SEM. Control vs. STNx-vehicle (disease effect) *P < 0.05, **P < 0.01; STNx-vehicle vs. ramipril (treatment effect) ^†^*P* < 0.05, ^††^*P* < 0.01

### Cardiac expression of KLF15 gene and protein is reduced in CKD rats and restored by ramipril treatment

There was a 50% reduction in cardiac KLF15 gene expression in STNx rats with LVH compared to Control rats, there (*P* < 0.05; Fig. 1b) with a corresponding fall in KLF15 protein (*P* < 0.05, Fig. 1c). Treatment with ramipril led to increases in both cardiac KLF15 mRNA (*P* < 0.05, Fig. 1b) and protein expression (*P* < 0.01, Fig. 1c).

**Fig. 2:**
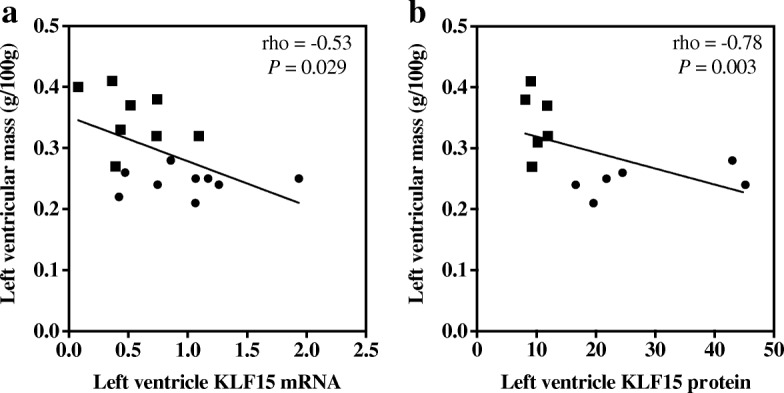
Correlation of left ventricular mass with KLF15 gene and protein expression. Left ventricular mass correlates negatively with both KLF15 mRNA (**a**) and protein (**b**) expression. Black circle = Control, black square = subtotal nephrectomy-Vehicle

**Fig. 3:**
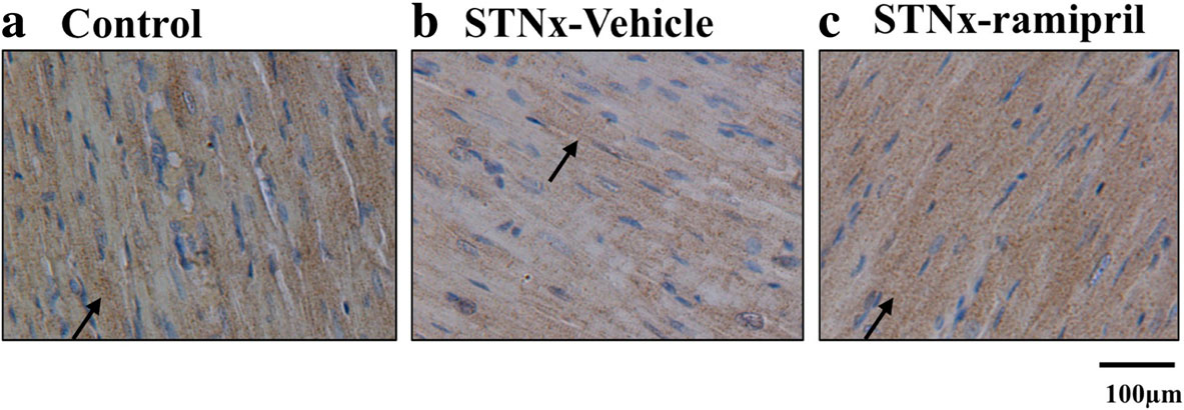
Representative immunohistochemistry in left ventricular sections stained for total KLF15 protein (brown staining). Blue staining shows the nuclei. Black arrows show the presence of KLF15 brown staining. Images taken at × 20 magnification, scale bar 100 μm. **a** Control; **b** STNx-Vehicle **c** STNx-ramipril. STNx, subtotal nephrectomy

There was a significant negative correlation between LV mass and LV KLF15 gene (rho = − 0.53, *P* = 0.029; Fig. 2a) and protein expression (rho = − 0.78, *P* = 0.003; Fig. 2b) when assessed in vehicle treated Control and STNx rats. KLF15 gene and protein expression was lowest in rats with the highest LV mass. Figure 3 shows KLF15 immunohistochemical staining in the cytoplasm and nuclei of myocytes, with reduced staining in STNx rats.

## Discussion

This study in experimental CKD induced LVH is the first evidence that imbalance between key regulators and mediators of hypertrophy and fibrosis, namely KLF15 and the RAS pathway occurs. In CKD, loss of cardiac KLF15 expression at the gene and protein level led to unchecked trophic and fibrotic signalling, with significant increases in the pro-hypertrophic transcriptional pathway, GATA4, as well as in several key markers of hypertrophy (ANP, BNP) and fibrosis (CTGF). ACE inhibitors are commonly used to lower blood pressure and regress LVH and fibrosis but to date their effect on KLF15 expression is unknown. In this study, ACE inhibition was associated with a significant increase in KLF15 gene and protein expression, and a corresponding reduction in cardiac expression of ANP, BNP and CTGF.

In vitro, KLF15 overexpression inhibits cell size and hypertrophic gene expression [11]. In vivo in rats, KLF15 expression is down regulated in response to pressure overload induced by trans-aortic constriction (TAC) [11], and in the Ren-2 model of hypertension induced LVH, loss of the constitutive presence of cardiac KLF15 precedes progression to heart failure [20]. KLF15 null mice are viable but develop cardiac hypertrophy and heart failure in response to TAC [11] and angiotensin II (Ang II) infusion [12]. However, neither of these studies showed the effects of KLF15 on LV mass or used intervention with drugs known to regress LVH [11, 12]. The observed effects were not related to differences in blood pressure between wild type and null mice [12]. In mice, adenoviral overexpression of KLF15 in the heart prevents the development of Ang II induced cardiac hypertrophy [21]. To our knowledge there are no further studies investigating other antihypertensive agents and their effect on cardiac KLF15 expression in experimental CKD. It is not known if KLF15 expression is directly influenced by ramipril independently of the fall in blood pressure, or if other antihypertensive agents would have the same effect. To address this, further studies are required in experimental CKD to examine the effect of various antihypertensive agents on KLF15 levels and if associated changes are independent of blood pressure.

Previous in vivo studies regardless of the experimental model used have consistently shown that the loss or reduction of KLF15 removes the ability to repress key cardiac transcription factors that enable growth contributing to the development of cardiac hypertrophy [22]. KLF15 represses cardiac hypertrophy in part through the modulation of the activity of GATA4 and MEF2, which are central mediators of hypertrophic remodelling acting through ANP and BNP. KLF15 inhibits GATA4 and MEF2 DNA-binding transcriptional activation by preventing their binding to transcriptional targets [11, 13]. Adenoviral overexpression of KLF15 in neonatal rat ventricular myocytes reduces ANP and BNP mRNA expression and strongly inhibits phenylephrine induced ANP and BNP promoter activity [11]. CTGF, a key mediator of fibrosis in pathological hypertrophy [23, 24], is negatively regulated by KLF15 [13]. Adenoviral KLF15 overexpression in neonatal rat ventricular fibroblasts inhibits both basal and transforming growth factor β induced CTGF expression and the activity of the CTGF promoter [13]. Although previous studies have shown direct effects of KLF15 on ANP, BNP, GATA4 and CTGF, direct studies were not performed in the current study and will be required in future studies investigating KLF15 in CKD.

Transgenic overexpression of GATA4 results in severe cardiomyopathy and early death in mice [25] and acute hemodynamic stress due to bilateral nephrectomy increases ventricular BNP reporter expression through a GATA4-dependent pathway [26]. Pressure or volume overload induced cardiac hypertrophy also leads to significant increases in MEF2 activity [27]. In the current study, we found an increase in GATA4 but not MEF2A gene expression in the hearts of rats with CKD, and a reduction in expression with ramipril although the changes were not significant. It is possible that the results reflect use of cardiac tissue homogenates rather than nuclear extracts, particularly as we saw major increases in cardiac ANP and BNP gene expression in STNx with a corresponding reduction with ramipril.

The pathogenesis of LVH is complex. It is characterised by myocyte hypertrophy and fibrosis which causes progressive impairment in cardiac contractility and increasing stiffness of the myocardium leading to diastolic and systolic dysfunction and eventually heart failure. Blood pressure reduction is the main treatment approach for patients with LVH, with some evidence that drugs that target the RAS have a specific effect on LVH, independent of blood pressure reduction [28]. However despite current drug therapies, patients with LVH and especially those with CKD induced LVH remain at high risk of CV complications including sudden death and heart failure [2].

In this paper we identify a potential role for cardiac KLF15 deficiency in the pathogenesis of CKD induced LVH. Our results have clinical relevance as there is evidence that loss of cardiac KLF15 may contribute to LVH and the progression to heart failure in humans. Patients with LVH secondary to aortic stenosis compared to those without LVH, have significantly reduced KLF15 protein expression in the nuclei of myocytes [11]. In our study we did not specifically quantitate KLF15 protein in the nuclei but found some KLF15 protein staining overlapping nuclei. In human myocardium sections quantitated for KLF15 using immunohistochemistry, a granular cytoplasmic KLF15 protein expression pattern similar to our study has been shown [29]. We are not aware of any other studies that have quantitated KLF15 protein using immunohistochemistry in cardiac tissue. In patients with non-ischemic cardiomyopathy, cardiac KLF15 gene expression was reduced by 50% compared to control patients [12], and in those undergoing a left ventricular assist device implantation and explantation as a bridge to transplantation, KLF15 was reduced in the failing heart compared to controls, with significant recovery of KLF15 expression after mechanical unloading [30].

To date, the studies of KLF15 in kidney disease have focused on its role in kidney fibrosis [31, 32]. In the STNx model of CKD, kidney KLF15 mRNA and protein expression were reduced in the remnant kidney, and dietary protein restriction increased KLF15 and reduced kidney fibrosis [31]. The authors also reported that overexpression of KLF15 in mesangial and HEK293 cells significantly reduced fibronectin and type IV collagen mRNA levels [31].

The KLFs can act as either transcriptional repressors or activators. Nine KLF members are expressed in neonatal rat myocytes after endothelin-1 stimulation, with KLF2, KLF4, KLF5, KLF6, KLF9 and KLF10 mRNA increasing after stimulation and reduced expression of KLF3, KLF11 and KLF15 [33]. Of these only KLF4, KLF5, KLF10, KLF11 and KLF15 have been studied in cardiac hypertrophy. In vitro, KLF4 expression is induced with hypertrophic stimulation with either Ang II, endothelin-1 and phenylephrine in neonatal rat ventricular myocytes [34]. In vivo, cardiac hypertrophy induced by either chronic Ang II infusion or TAC results in significant increased cardiac KLF4 gene expression [34]. KLF4 is also expressed in Ang II stimulated cardiac fibroblasts and KLF4 overexpression results in increased collagen mRNA expression [35]. Ang II infusion in heterozygous KLF5 knockout mice resulted in reduced cardiac hypertrophy and interstitial fibrosis compared with wild type mice [36]. In another study, male KLF10 knock out mice developed cardiac hypertrophy compared to wild type mice [37]. KLF11 expression is reduced in hypertrophic mouse hearts and overexpression of cardiac KLF11 protected mice from cardiac hypertrophy induced with TAC [38]. The current study focussed on the change in expression of KLF15, and future studies that examine the concomitant expression of several KLFs in cardiac hypertrophy may be informative.

## Conclusion

In summary, experimental CKD induced LVH is associated with loss of cardiac KLF15 expression, and activation of hypertrophic and fibrotic pathways. We provide the first evidence that drugs commonly used in the clinic, namely ACE inhibitors, reduce blood pressure and LVH and restore cardiac KLF15 expression. Studies are now needed to determine if this is a specific effect of ACE inhibition. Further studies are also required to determine whether other anti-hypertensive agents that reduce blood pressure and LVH would also be associated with changes in cardiac KLF15 levels in CKD. In the future, approaches that directly elevate cardiac KLF15 may emerge as an important therapeutic option to halt the CV complications of CKD.

## Abbreviations

ACE: Angiotensin converting enzyme
Ang II: Angiotensin II
ANP: Atrial natriuretic peptide
BNP: Brain natriuretic peptide
CKD: Chronic kidney disease
CTGF: Connective tissue growth factor
CV: Cardiovascular
GATA4: GATA binding protein 4
KLF15: Kruppel-like factor 15
LV: Left ventricle
LVEDP: Left ventricle end-diastolic pressure
LVH: Left ventricular hypertrophy
MAP: Mean arterial pressure
MEF2: Myocyte enhancer factor 2 mRNA
mRNA: Messenger ribonucleic acid
RAS: Renin angiotensin system
STNx: Subtotal nephrectomy
TAC: Transverse aortic constriction
